# Refractory Hypotension as an Initial Presentation of Bilateral Subclavian Artery Stenosis

**DOI:** 10.1155/2016/8542312

**Published:** 2016-07-28

**Authors:** Maxwell Eyram Afari, John V. Wylie, Joseph P. Carrozza

**Affiliations:** Division of Cardiology, St. Elizabeth's Medical Center, Tufts University School of Medicine, Brighton, MA 02135, USA

## Abstract

Bilateral subclavian stenosis is a rare clinical condition. An interbrachial pressure difference of 15 mm Hg can raise suspicion for unilateral subclavian artery stenosis, but the diagnosis of bilateral subclavian artery stenosis can be challenging. We present a case of a 75-year-old woman who presented with refractory hypotension after surgery. Initial vitals revealed blood pressure in the 60s/50s mm Hg in both arms. Cardiopulmonary examination was remarkable for diminished pulses in all 4 extremities and audible carotid bruits. She continued to be hypotensive despite aggressive fluid resuscitation. Troponin T peaked at 0.24 ng/mL (reference < 0.04), and an echocardiogram revealed a reduction in ejection fraction (37% from 50%). Left and right heart catheterization demonstrated normal filling pressures and cardiac output. During the procedure, however, it was noted that the patient's central blood pressure was 70–80 mm Hg higher than cuff pressures obtained in either arm. Selective angiography revealed 90% left subclavian ostial stenosis as well as 70% stenosis of the right subclavian artery.

## 1. Introduction

Bilateral subclavian stenosis is a rare clinical condition [1]. In most clinical settings, unilateral brachial blood pressure is commonly relied upon to make clinical decisions. Rarely do clinicians check both brachial blood pressure values or check even one lower extremity blood pressure. We report a case of an elderly woman who presented with refractory hypotension and was found to have bilateral subclavian artery stenosis.

## 2. Case

A 75-year-old woman with multiple medical problems was found to be hypotensive after transurethral bladder tumor resection. Her past medical history was remarkable for coronary artery disease with a drug-eluting stent to the left anterior descending artery 5 years ago, chronic diastolic congestive heart failure, hypertension, hyperlipidemia, and diabetes mellitus. Past surgical history included left carotid endarterectomy and bilateral femoral bypass 10 and 8 years ago, respectively. After the patient's bladder tumor resection, she was noted to be persistently hypotensive but asymptomatic. Physical examination revealed a well-appearing woman in no acute distress, afebrile, with blood pressure of 66/50 mm Hg (right arm), oxygen saturation of 90% on 3 liters of oxygen per nasal cannula, respiration rate of 20 per minute, and heart rate of 96 beats per minute. Cardiopulmonary examination was only remarkable for a bruit in the bilateral carotid region. Jugular venous distension and peripheral edema were absent. Mild tenderness of the abdomen was noted.

**Figure 1:**
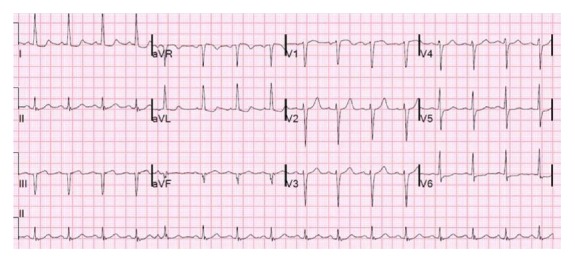
Electrocardiogram showed sinus tachycardia, evidence of old inferior and anterior infarct, left ventricular hypertrophy, and nonspecific lateral ST abnormalities.

The patient was aggressively resuscitated with three liters of normal saline with no improvement in her blood pressure. Blood pressure checked in the contralateral arm was similar. Electrocardiogram showed poor R-wave progression, evidence of old inferior infarct (Q waves), left ventricular hypertrophy, and nonspecific lateral ST abnormalities (Figure 1). The peaked cardiac enzymes were troponin T 0.24 ng/mL (reference <0.04), creatine kinase-myocardial band 10.1 ng/mL (reference < 6.0), and creatine phosphokinase 121 U/L (reference 38–234). An echocardiogram estimated an ejection fraction of 37% (down from 50% a year prior to admission), diastolic dysfunction, no valvulopathy, and apical and septal hypokinesis. Computed tomography angiogram ruled out pulmonary embolism and demonstrated calcification of the coronary arteries and the aorta.

**Figure 2:**
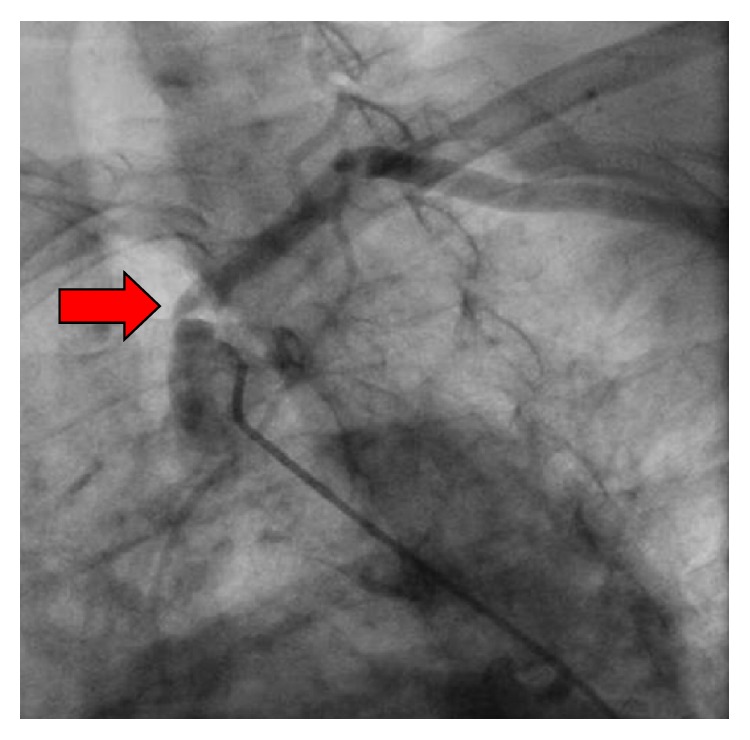
Selective angiography of the left subclavian artery revealed 90% ostial stenosis.

**Figure 3:**
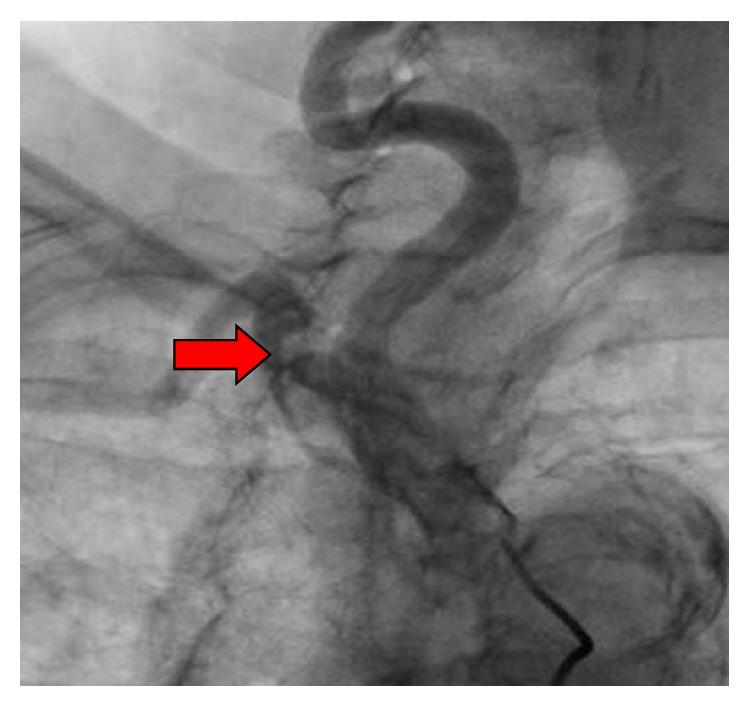
Selective angiography of the right brachiocephalic artery revealed 70% stenosis of the right subclavian artery.

Based on the results above, the patient was sent for cardiac catheterization to rule out new coronary obstruction. The previous LAD stent was found to be patent and the previously noted occluded RCA with collaterals was also unchanged. During the procedure, her central blood pressure was noted to be 204/67 which was 70–80 mm Hg higher than cuff pressure values obtained in either arm. Selective angiography of the left subclavian artery revealed 90% ostial stenosis (Figure 2) while selective angiography of the right brachiocephalic artery revealed 70% stenosis of the right subclavian artery (Figure 3).

## 3. Discussion

Subclavian artery (SCA) stenosis is defined by angiographic finding of >50% stenosis in the subclavian arteries. The incidence of SCA stenosis is estimated at approximately four percent [2]. A relationship between SCA stenosis, age, smoking, HDL cholesterol, and the presence of peripheral artery disease has been established [1]. Independent of cardiovascular risk factors and baseline cardiovascular disease, the presence of subclavian stenosis predicts mortality [3].

The most common presentation of subclavian stenosis is “subclavian steal syndrome.” This refers to the reversal of flow in a branch of the subclavian artery due to proximal stenosis of SCA. This reversal of flow can occur in an internal mammary artery graft and thus trigger symptoms of cardiac ischemia [4]. Alternatively, patients may present with dizziness, diplopia, ataxia, or syncope due to the reversal of blood flow in the vertebral artery in order to “steal blood from the brain” [5]. Invariably, patients will present with signs of ischemia in the upper extremities including claudication, muscle fatigue, or finger necrosis. Hypotension is a rare presentation of subclavian stenosis with very few literature reports [5–7].

Diagnosis of SCA stenosis requires a high index of clinical suspicion in patients with symptoms of coronary subclavian steal syndrome or vertebrobasilar insufficiency. The measurement of bilateral brachial blood pressure should be the first evaluation if this condition is suspected. A difference of over 15 mm Hg is considered significant and is suggestive of unilateral subclavian stenosis, with sensitivity and specificity of 50% and 90%, respectively [8]. Consensus guidelines on the management of arterial hypertension recommend the evaluation of blood pressure between both arms [9, 10]. As shown in our case, bilateral subclavian artery stenosis can be missed when the blood pressure measurement is similar between both arms. This raises the question of how many asymptomatic bilateral subclavian stenosis cases we are missing in clinical practice.

In select patients, we believe that the comparison of upper extremity blood pressure to lower extremity blood pressure can aid in the diagnosis. Unfortunately, obtaining lower extremity blood pressure is not a routine clinical practice. Our patient had chronic peripheral vascular disease which equally distorted her lower extremity blood pressure and made the diagnosis even more challenging. Delayed or decreased amplitude pulses and bruits in the subclavicular fossa are physical findings which could be a clue to SCA stenosis.

Our initial diagnostic differential diagnosis included sepsis, pulmonary embolism, cardiac tamponade, endocrine etiologies such as hypothyroidism or hypoadrenalism, abdominal compartment syndrome, and Takayasu arteritis. Based on the elevated cardiac enzymes and the newly decreased ejection fraction, concern for perioperative myocardial infarction was on top of our differential diagnosis, hence the cardiac catheterization. Though angiogram is the gold standard to determine vascular stenosis, the diagnosis of SCA stenosis can be made through noninvasive imaging modalities such as duplex ultrasound, continuous flow Doppler, computed tomography angiography, and magnetic resonance angiography. Though our patient did have a CTA, the primary indication was to perform assessment for pulmonary embolism rather than imaging the subclavian arteries.

Subclavian artery stenosis is associated with increased cardiovascular and overall mortality [3]. In projecting data from peripheral artery disease, it is suspected that patients with SCA stenosis benefit from aggressive secondary prevention. We encouraged our patient to continue aspirin, statin, beta-blocker, and angiotensin-converting enzyme inhibitor, all of which she was already taking due to her history of coronary artery disease. She was also counselled on smoking cessation and weight loss.

There is no data to support preemptive revascularization in asymptomatic patients. Schillinger et al. compared medical therapy to percutaneous transluminal angioplasty of SCA stenosis in terms of long-term hemodynamic and symptomatic outcome [11]. The risk of symptomatic stenosis was found to be nonsignificant at 42-month follow-up. However, it should be noted that medical therapy has improved significantly since the study. In patients with symptomatic subclavian artery stenosis, angioplasty plus stenting was found to be superior to angioplasty alone or bypass in terms of asymptomatic survival and freedom from reintervention [12].

This case highlights the importance of having a high index of suspicion for bilateral subclavian artery stenosis in a patient presenting with refractory hypotension and known peripheral vascular disease. As we highlighted above, pseudohypotension could mask clinically significant hypertension and this could contribute to an elevated risk of cardiovascular events.
